# Microfluidics for simultaneous quantification of platelet adhesion and blood viscosity

**DOI:** 10.1038/srep24994

**Published:** 2016-04-27

**Authors:** Eunseop Yeom, Jun Hong Park, Yang Jun Kang, Sang Joon Lee

**Affiliations:** 1 School of Mechanical Engineering, Pusan National University, Busan, South Korea; 2 Department of Mechanical Engineering, Pohang University of Science and Technology (POSTECH), Pohang, South Korea; 3 Department of Mechanical Engineering, Chosun University, Gwangju, South Korea

## Abstract

Platelet functions, including adhesion, activation, and aggregation have an influence on thrombosis and the progression of atherosclerosis. In the present study, a new microfluidic-based method is proposed to estimate platelet adhesion and blood viscosity simultaneously. Blood sample flows into an H-shaped microfluidic device with a peristaltic pump. Since platelet aggregation may be initiated by the compression of rotors inside the peristaltic pump, platelet aggregates may adhere to the H-shaped channel. Through correlation mapping, which visualizes decorrelation of the streaming blood flow, the area of adhered platelets (*A*_*Platelet*_) can be estimated without labeling platelets. The platelet function is estimated by determining the representative index *I*_*A·T*_ based on *A*_*Platelet*_ and contact time. Blood viscosity is measured by monitoring the flow conditions in the one side channel of the H-shaped device. Based on the relation between interfacial width (*W*) and pressure ratio of sample flows to the reference, blood sample viscosity (*μ*) can be estimated by measuring *W*. Biophysical parameters (*I*_*A·T*_, *μ*) are compared for normal and diabetic rats using an *ex vivo* extracorporeal model. This microfluidic-based method can be used for evaluating variations in the platelet adhesion and blood viscosity of animal models with cardiovascular diseases under *ex vivo* conditions.

Blood is a concentrated suspension of red blood cells (RBCs), leukocytes, platelets, and protein macromolecules in plasma. Among these blood elements, platelets constitute a key component of normal hemostasis and pathological thrombosis^1^. A multistep adhesion process between platelet membrane receptors and adhesive ligands enables cell adhesion efficiently under dynamic flow conditions. Subendothelial matrix proteins^2^, biochemical activators^3^,^4^ and hemodynamic features^5^,^6^,^7^ contribute to this adhesion mechanism. High shear conditions, induced by arterial narrowing, establish a potentially hazardous cycle of further platelet activation and thrombus growth^8^. In addition, the platelet activation plays a critical role in cardiovascular diseases including arteriosclerosis, stroke, and diabetes mellitus^1^,^9^,^10^.

The mechanisms underlying platelet aggregation and thrombus formation vary with shear rate conditions to some extent. Under a relatively low shear condition that ranges from 0 to 1,000 s^−1^, platelet aggregation is primarily influenced by soluble fibrinogen^11^,^12^. Fibrinogen can support both adhesion and aggregation of platelets by binding to glycoprotein (GP) IIb/IIIa receptor and integrin α_IIb_β_3_^4^. At progressively high shear conditions, the fibrinogen plays a supportive role and von Willebrand factor (vWF) becomes more prominent^11^. The GPIb on the platelet engages in vWF^13^. When the shear rate is higher than 10,000 s^−1^, the aggregation mechanism becomes exclusively influenced by the vWF engagement of GPIb^14^.

Several methods and devices are introduced to measure platelet functions^7^,^15^,^16^,^17^,^18^,^19^. A light transmission aggregometry (LTA) uses platelet-rich plasma (PRP) for testing platelet functions^16^,^20^. However, LTA is time-consuming and difficult to standardize the platelet function. Given these reasons, alternative methods such as impedance aggregometry, rapid platelet function analyzer (RPFA), platelet function analyzer 100 (PFA-100, Siemens, Germany), 96-well plate aggregometry, and flow cytometry were introduced to measure platelet aggregation in PRP or whole blood^18^,^21^. Although impedance aggregometry estimates the degree of platelet aggregation by measuring variations in electrical resistance^22^,^23^, much sample volume is required and the analysis is time-consuming and expensive. The RPFA developed as a point-of-care instrument is based on monitoring integrin α_IIb_β_3_ (GPIIb/IIIa)^24^. However, the measurement performance of this aggregometry is improved by the presence of fibrinogen-coated beads. The PFA-100 is composed of a sample reservoir, a capillary, and a biologically active membrane with a central aperture (collagen-epinephrine-coated or collagen-ADP-coated). A test sample is delivered from the reservoir through the capillary and the aperture under high shear rate conditions (5000–6000 s^−1^). For quantification of platelet aggregation, the time which is necessary for a platelet plug to occlude the aperture is monitored^25^. However, this method has limitation of sensitivity and specificity^18^,^26^. Although the platelet aggregation can be measured using 96-well plate reader with relatively low sample volume^27^, preparation of PRP sample is required for measurement. A flow cytometry assay was used to assess the platelet function from small volume of blood with low platelet numbers^28^,^29^. However, this is quite an elaborate technique because blood sample should be divided into two parts by labeling platelets with different biomarkers, washing away the excess of antibodies, and then reconstituting cells in a buffer solution with plasma prior to the actual assessment^21^,^30^.

Microfluidic systems can measure the functions of platelets in whole blood with or without using biomarkers^31^,^32^. Especially, it is demonstrated that the microfluidic system can provide diagnostic information related to hemostatics^18^. For these reasons, several microfluidic systems have been proposed using small sample volume^5^,^6^,^30^,^33^,^34^,^35^. In most previous studies, platelet aggregation is estimated by quantifying adhesion of platelet by changes in shear rate distribution. Besides these measurements, a rotating stirrer was placed inside a channel to induce platelet aggregation^7^. However, platelet function measured under *in vitro* conditions can be different from the results obtained under *in vivo* or *ex vivo* results to some extent^36^.

Blood viscosity is a representative parameter describing hemorheological characteristics. Fibrinogen concentration acting on platelet aggregation is closely associated with changes in blood viscosity. For understanding relations between cardiovascular diseases and hemorheological properties, a simultaneous measurement of platelet function and blood viscosity is important. The flow-switching phenomenon in an H-shaped microfluidic device, which is composed of two parallel side channels and a bridge channel, was monitored in our previous studies to investigate the relationship between blood viscosity and hemodynamic features under various physiological conditions^37^,^38^,^39^. The flow-switching phenomenon in the bridge channel occurs based on hydrodynamic force balancing between two parallel side channels. When the pressures at the both side ends of the bridge channel become the same, the flow in the bridge channel is not observed. The blood viscosity can be estimated from the ratio of flow rate of the blood and PBS solution at the hydrodynamic balancing state.

Platelet adhesion and blood viscosity are simultaneously measured by modifying a experimental means considerably using the H-shaped channel in the present study. The measurement performance of this microfluidic technique is examined using PRP and whole blood samples. Finally, the changes in these biophysical parameters between normal and diabetic rats are compared by using an extracorporeal model^39^,^40^.

## Simultaneous Estimation of Platelet Adhesion and Blood Viscosity

**Figure 1:**
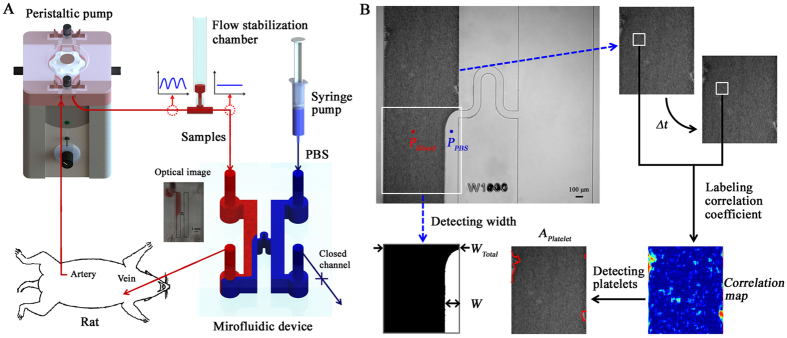
Proposed microfluidic-based method for measuring platelet function and blood viscosity. (**A**) Schematic of the measurement system composed of a peristaltic pump, a flow stabilization chamber, and a microfluidic device. An optical image of the microfluidic device is inserted. The outlet of the PBS flow is closed to induce an interfacial line between the PBS solution and blood sample. Blood is supplied by a rat extracorporeal loop. The blood and PBS solution mixture is returned to the jugular vein of the rat model. The drawings of peristaltic pump, syringe and microfluidic device were created by the authors using SolidWorks software (Dassault Systèmes SolidWorks Corp., USA). (**B**) Data processing; a correlation map labeling the square of the 2D correlation coefficient in the tiles of two consecutive images is obtained to quantify the area of adhered platelet (*A*_*Platelet*_). After a thresholding technique is applied, the adhered platelets can be distinguished from the streaming blood flow. The ratio of interfacial width of the PBS solution and total width of side channel (*W/W*_*Total*_) is measured using a thresholding technique to determine blood viscosity.

Since platelet aggregates formed by the peristaltic pump can easily occlude the narrow bridge channel, it is technically difficult for the experimental methods used in our precious studies for viscosity measurement^37^,^38^ to monitor the hydrodynamic balancing state. To estimate blood viscosity without flow occlusion, the present method introduces an interfacial line between the PBS solution and the test blood sample by blocking the outlet of one side-channel used for PBS solution. In order to prevent the adhesion of platelets in the region near the interfacial line, the region of interest (ROI) used for viscosity measurement is incubated with 2% bovine serum albumin (BSA, Sigma, MO) for 10 min. Based on these modifications in experimental means, platelet adhesion and blood viscosity can be estimated simultaneously. As illustrated in Fig. 1A, a rat extracorporeal model is employed to monitor temporal variations in platelet adhesion and blood viscosity more accurately^38^. The pulsatile flow generated by a peristaltic pump is stabilized by passing through a flow stabilization apparatus.

To measure blood viscosity (*μ*), pressures for blood (*P*_*Blood*_) and PBS solution (*P*_*PBS*_) are estimated based on the location of the interfacial line (Fig. 1B). Pressure in the microchannel (*P*) can be expressed by the following equation;


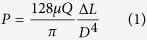


where, *L* and *D* are the characteristic length and hydraulic diameter of the microchannel, respectively. *Q* indicates the flow rate. Considering that an interfacial width (*W*) of the PBS solution in the left side channel can be determined based on the pressure ratio, the blood viscosity (*μ*_*Blood*_) can be estimated using the following equation;


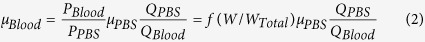


where *μ*_*PBS*_ is the viscosity of PBS solution (1 cP). *Q*_*Blood*_ and *Q*_*PBS*_ are flow rates of blood and PBS solution, respectively. *f*(*W*/*W*_*Total*_) is a function of the normalized interfacial width divided by the total width of the side channel. The interfacial line between the blood and PBS solution is detected through an image-processing technique based on thresholding.

The rotors of the peristaltic pump compress the flexible tube to pump blood. Considering that high shear force can initiate platelet aggregation, this kind of occlusion in the peristaltic pump may induce platelet aggregation. Then platelets easily adhere to the microchannel. However, it is difficult to distinguish adhered platelets from flowing blood in the captured optical images. A correlation map, proposed in our previous study, could depict the degree of decorrelation of flowing blood by labeling the 2D correlation coefficient (*R*) between the small tiles of two consecutive images with a time interval of Δ*t*^41^. Each image is divided into *m* × *n* small tiles. The coefficient R of the tiles is calculated by using the following equation:


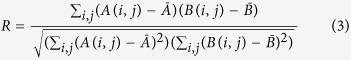


where, *A* and *B* represent the tiles of two consecutive images, (*i, j*) is the pixel coordinate of the tile, and 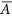 and 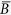 denote the mean values of tiles *A* and *B*, respectively. The size of tile used in this study is 11 ×11 pixels. A squared correlation coefficient is labeled at the centroid of the tile with a color scale. After labeling a specific pixel, the labeling process is repeated for all pixels. Since the correlation coefficients around adhered platelets are significantly high, the regions of adhered platelets are more clearly distinguished in the correlation map. After applying the thresholding technique with an optimal value of Otsu’s algorithm, the correlation maps are converted into binary images. The area of the adhered platelets is estimated by counting the number of nonzero pixels in the binary image.

This estimation method has several extraordinary advantages. First, the biophysical properties, including blood viscosity and platelet adhesion, can be measured simultaneously. Second, labeling of platelet or other blood cells is not required. Third, the method can measure the biophysical properties of blood circulating in the extracorporeal microfluidic loop without collecting blood sample from a rat model. Since the hemorheological properties are affected by external exposure of blood samples^42^, *ex vivo* measurement is important for their accurate estimation without remarkable hemorheological changes. The present estimation method allows quick and easy operation since the peristaltic pump is utilized as means to induce platelet aggregation.

## Results and Discussion

### Pressure ratio estimation

**Figure 2:**
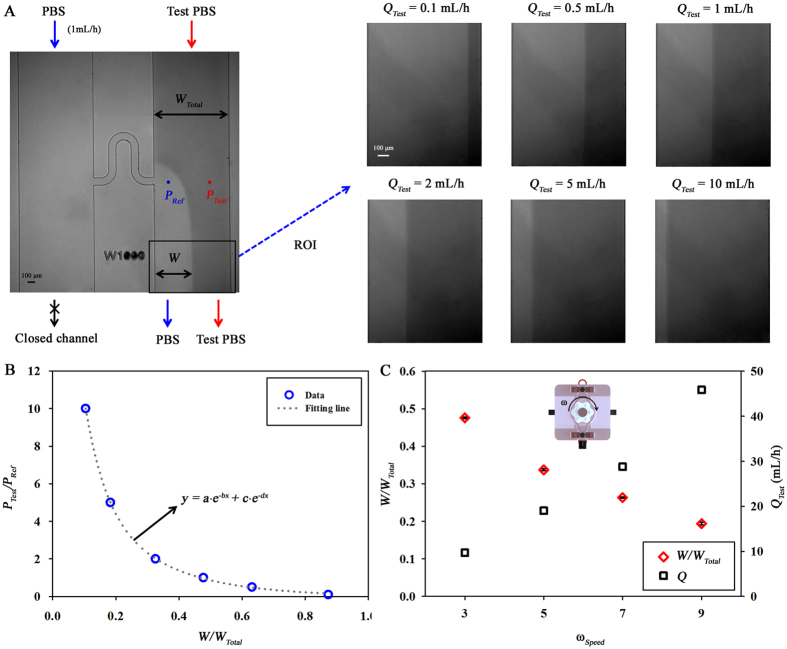
Relation between interfacial width and pressure ratio of PBS solution and test fluid. (**A**) Flow image of PBS and test PBS solutions. The outlet of PBS flow is closed to induce an interfacial line between the PBS and test PBS solutions. Magnified images represent variations in the interfacial width of PBS solution (*W*) in the region of interest according to the flow rate of the test PBS (*Q*_*Test*_ = 0.1–10 mL/h). (**B**) Variation in the pressure ratio between the PBS and test PBS solutions according to the normalized interfacial width (*W/W*_*Total*_). A fitting curve equation is determined by conducting regression analysis. (**C**) Variations in the *W/W*_*Total*_ ratio and flow rate (*Q*_*Test*_) according to rotational speed unit of the peristaltic pump (*ω*_*Speed*_). The flow rate of PBS solution is set to 10 mL/h. The drawing of peristaltic pump was created by the authors using SolidWorks software. Each dot indicates the mean value obtained from three samples.

For viscosity estimation, a pressure condition in the H-shaped channel should be accurately monitored. The PBS and test PBS labeled with Rhodamine B dye at a low concentration (0.0125%) flow into the microchannel by syringe pumps to investigate interaction between pressure and interfacial width (W). For that, the reference PBS solution is fixed at a flow rate of 1 mL/h and the flow rate of test PBS (*Q*_Test_) is varied from 0.1 to 10 mL/h. The viscosity of PBS labeled with Rhodamine B is the same as that of the PBS solution. Figure 2A shows flow condition for the PBS and test PBS solutions according to *Q*_*Test*_. The width becomes narrower as *Q*_*Test*_ increases. Since viscosity of PBS and test PBS is the same, pressure ratio of test PBS to PBS (*P*_*Test*_*/P*_*Ref*_) is the flow rate ratio (*Q*_*Test*_/*Q*_*Ref*_) of them. As shown in Fig. 2B, the pressure ratio (*P*_*Test*_*/P*_*Ref*_) increases rapidly as the interfacial width ratio (*W*/*W*_*Total*_) decreases from the value of 0.3. Following fitting equation is adopted for estimation of *P*_*Test*_*/P*_*Ref*_ using *W*/*W*_*Total*_;


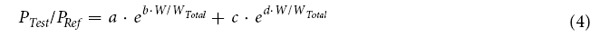


Four fitting parameters are determined through regression analysis (a = 25.6, b = −15.6, c = 7.9, d = −4.4).

By using Eq. (4), the unknown flow rate of the test PBS solution can be estimated according to the rotational speed unit of the peristaltic pump (*ω*_*Speed*_). For accurate estimation of the flow rate delivered by the peristaltic pump, the reference PBS solution is supplied at a flow rate of 10 mL/h in the consideration of high flow rate of the peristaltic pump. Figure 2C shows variations in *W*/*W*_*Total*_ and *Q*_*Test*_ as a function of *ω*_*Speed*_. The *Q*_*Test*_ increases with decline in *W*/*W*_*Total*_ as *ω*_*Speed*_ becomes higher as expected: *Q*_*Test*_ = 9.7, 19.0, 28.8, and 45.9 mL/h at *ω*_*Speed*_ of 3, 5, 7, and 9, respectively. To identify the pulsatile flow features induced by the peristaltic pump, a micro particle image velocimetry (PIV) technique was employed. Fast Fourier transform (FFT) was applied to the measured flow rate information. Peak frequencies of the pulsatile flow are 0.5865, 1.0401, 1.6129 and 2.2796 Hz at *ω*_*Speed*_ = 3, 5, 7, and 9, respectively. By comparing the flow rates measured by the interfacial width ratio and the flow rate measured by micro-PIV technique, the performance of the proposed method was validated. As depicted in Supplementary Fig. 1, the flow rates estimated by the interfacial width ratio are in a good agreement with the flow rates measured by micro-PIV technique.

### Platelet adhesion and viscosity of PRP

**Figure 3:**
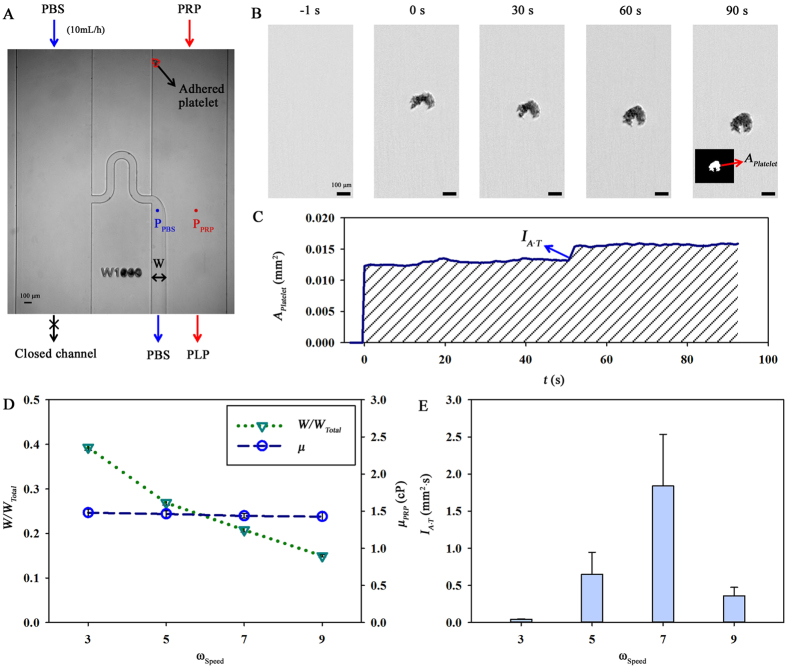
Measurement of platelet adhesion and viscosity by using platelet-rich plasma (PRP). (**A**) Typical flow image of PBS solution and PRP. The outlet of PBS flow is closed to induce an interfacial line between the PBS solution and PRP. (**B**) Magnified images represent the progress of platelet adhesion with time. Scale bar indicates 100 μm. A binary image showing the platelet area (*A*_*Platelet*_) is inserted. (**C**) Temporal variation in *A*_*Platelet*_ for the platelets in image (**B**). An index related to platelet area and contact time (*I*_*A·T*_) is proposed to quantify platelet adhesion. (**D**) Variations in the normalized interfacial width (*W/W*_*Total*_) and viscosity of PRP (*μ*_*PRP*_) according to rotational speed unit of the peristaltic pump (*ω*_*Speed*_). (**E**) Comparison of *I*_*A·T*_ with regard to *ω*_*Speed*_. Each data set is obtained from three samples.

PRP has been widely used for the estimation of the platelet function because PRP is relatively easy to distinguish platelet adhesion compared with other samples^20^,^43^. As shown in Fig. 3A, the PBS solution and the PRP are flowed into the microfluidic device. Platelet aggregation in the PRP are initiated by high shear force as it passes through the peristaltic pump. Some platelet aggregates adhere to the side channel (indicated with a red line). Because the outlet of the left side channel is closed, the interfacial line between the PBS solution and the PRP is clearly visible in the right side channel.

Since collagen is coated on the glass substrate, platelet aggregates can adhere to every position on the H-shaped channel. Figure 3B shows temporal changes of platelet adhesion with the lapse of time. In the initial stages of platelet adhesion, the cell sticking on the wall supports this platelet adhesion. Since the tethered cells are subjected to hydrodynamic drag force, their rolling or translocation phenomena over the wall are observed^44^. It is more likely that adhered platelet layer accelerates clustering platelets and sustaining of cell adhesion^45^. Thus, running platelet aggregates are easily attached near the adhered platelet layer. Figure 3C shows the increase of adhered platelet area (*A*_*Platelet*_) with the lapse of time. An index related to the platelet area and contact time (*I*_*A·T*_) is evaluated using following mathematical equation to quantify platelet adhesion.


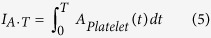


where, *T* indicates the measurement time duration (120 s in the present analysis). Measurement time is determined to minimize PBS injection into the rat model.

Variations of platelet adhesion and blood viscosity are investigated at different values of *ω*_Speed_ using PRP. As *ω*_*Speed*_ increases, *W*/*W*_*Total*_ decreases (Fig. 3D). By inserting Eq. (4) into *f*(*W*/*W*_*Total*_) of Eq. (2), the viscosity can be estimated according to *ω*_*Speed*_. However, the viscosity of the PRP (*μ*_*PRP*_) is almost constant, regardless of *ω*_*Speed*_ like the Newtonian fluid. This result may be attributed to the low concentration of platelets and leukocytes in PRP. Figure 3E shows variation in *I*_*A·T*_ with respect to *ω*_*Speed*_. Since the platelet aggregation is influenced by shear force^11^,^12^,^13^,^14^, *I*_*A·T*_ increases gradually with *ω*_Speed_. However, the index *I*_*A·T*_ decreases significantly at *ω*_*Speed*_ = 9 from *ω*_*Speed*_ = 7. This decline may result from that considerably high hydrodynamic drag force acting on adhered platelet induces platelets to detach.

### Effect of hematocrit on platelet adhesion and blood viscosity

**Figure 4:**
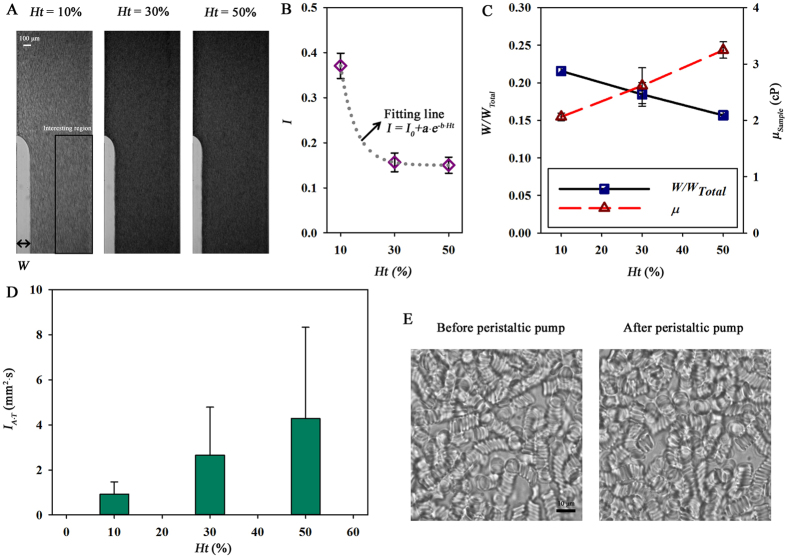
Variations in platelet adhesion and blood viscosity according to hematocrit (*Ht*). (**A**) Flow images of blood samples with three different *Ht* levels (*Ht* = 10%, 30%, and 50%). The interfacial line between PBS solution and blood samples is clearly shown by closing the outlet of PBS flow. (**B**) Variation in image intensity (*I*) according to *Ht*. *I* is obtained by averaging image intensity in the ROI (half of the downstream side channel). A fitting curve is overlapped with the data points. (**C**) Variations in the normalized interfacial width (*W/W*_*Total*_) and viscosity of blood sample (*μ*_*Sample*_) according to *Ht.* (**D**) Quantitative variation in the representative index that estimates platelet adhesion (*I*_*A·T*_) with regard to *Ht.* (**E**) Microscopic images of blood samples before and after passing through the peristaltic pump. Each data set is obtained from three samples.

Although the estimation method can accurately measure platelet function by using PRP, the bleeding time is inversely proportional to hematocrit^46^. Blood viscosity significantly varies with hematocrit. Therefore, the measurement of platelet adhesion and blood viscosity by utilizing whole blood is very important to investigate hematocrit effects. Three different hematocrit levels (*Ht* = 10, 30, 50%) are prepared for simultaneous measurement of platelet function and blood viscosity. Figure 4A shows optical images of blood sample with different hematocrit levels. For this experiment, a PBS solution flows from the opposite side channel to the test channel by passing through the bridge channel (*Q*_*PBS*_ = 10 mL/h). The flow rate of the test samples is fixed by setting *ω*_*Speed*_ to be 5. As the hematocrit level increases, blood sample becomes darker and the width of the PBS solution in the side channel decreases. For detailed analysis, normalized image intensity (*I*) is obtained by averaging the intensity profile in the region of interest (ROI) depicted in Fig. 4A. As depicted in Fig. 4B, *I* is dramatically reduced with increasing hematocrit level by up to 30%. Variations of image intensities between hematocrits of 30 and 50 is not clear. To examine the interaction between hematocrit and the image intensity, a fitting curve is derived (*I* = *I*_*0*_ + *a·e*^−*b·Ht*^). Three fitting parameters determined by regression analysis are *I*_*0*_ = 0.15, a = 1.29, b = −0.18.

The width ratio (*W*/*W*_*Total*_) inearly decreases against *Ht* (Fig. 4C). As expected, the viscosity of blood sample with a high hematocrit is higher than that with relatively low hematocrit. For investigation of the platelet function, a variation in *I*_*A·T*_ with respect to *Ht* is measured by applying the correlation map method (Fig. 4D). As the hematocrit level becomes high, the probability of platelet adhesion increases and adhered platelets are well sustained. When hematocrit level is high, platelets could be easily collided with other platelets or RBCs. This frequent collision of platelets may increase the shear force initiating platelet aggregation. Since the occlusion of a tube inside a peristaltic pump can change hemorheological properties, the effect of the peristaltic pump is examined. Figure 4E compares optical images of a blood sample with 50% hematocrit before and after passing through the peristaltic pump.

### Validation of a proposed method

**Figure 5:**
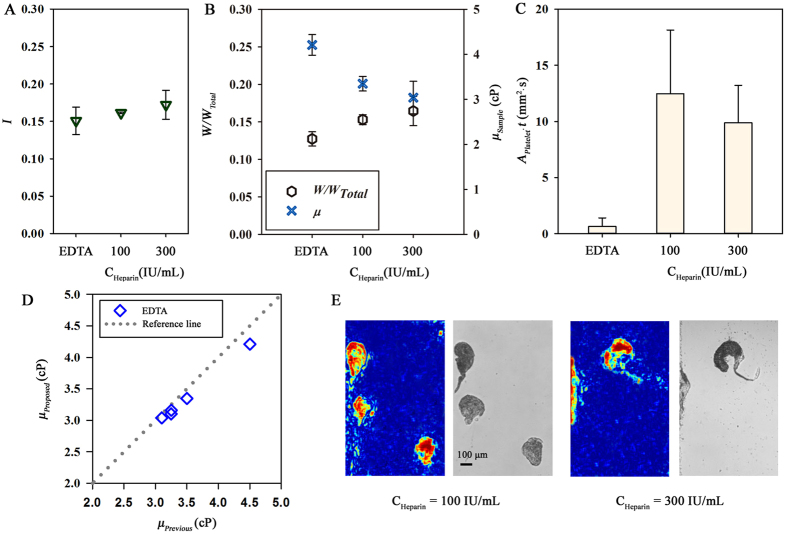
Validation of a proposed method. (**A**) Variation in the image intensities (*I*) of three blood samples anticoagulated with EDTA and different concentrations of heparin (*C*_*Heparin*_ = 100 and 300 IU/mL). (**B**) Variations in the interfacial width ratio (*W/W*_*Total*_) and viscosity of blood samples (*μ*_*Sample*_) anticoagulated with EDTA and heparin. (**C**) Quantitative comparison of representative index (*I*_*A·T*_) that estimates the platelet adhesion of blood samples anticoagulated with EDTA and heparin. Each data set is obtained from three samples. (**D**) Comparison of blood viscosities measured by the proposed method and previous method for blood samples anticoagulated with EDTA. (**E**) Representative correlation maps and optical images in the microfluidic device at heparin concentrations of 100 and 300 IU/mL.

For *ex vivo* measurement, blood coagulation inside the extracorporeal conduits should be prevented by the heparin injection^47^. However, the heparin injection can change various hemorheological properties such as hematocrit, and blood viscosity^48^,^49^,^50^. Therefore, the effects of heparin on the platelet adhesion, viscosity and hematocrit are investigated using blood samples anticoagulated by EDTA and heparin at different concentrations. Concentrations of heparin (*C*_*Heparin*_) are adjusted to be 100 and 300 IU/mL. Due to decrease of the hematocrit, the image intensity (*I*) increases, as the *C*_*Heparin*_ increases (Fig. 5A). As expected, the viscosity of blood sample (*μ*_*Sample*_) is reduced with increasing *C*_*Heparin*_ (Fig. 5B). The decline of *μ*_*Sample*_ at a high *C*_*Heparin*_ condition may be mainly influenced by the dilution effect. Besides the hematocrit and viscosity, the degree of platelet adhesion varies with *C*_*Heparin*_ (Fig. 5C). As the *C*_*Heparin*_ increases from 100 to 300 IU/mL, the *I*_*A·T*_ slightly decreases. Especially, *I*_*A·T*_ of the blood sample treated by heparin has a significantly higher value compared to that of the blood sample treated by EDTA. This tendency may be associated with inhibition of platelet response for EDTA treatment. By contrast, several other activities of platelets proceed despite the heparin injection^50^.

To validate the proposed method, viscosities measured by the proposed method are compared with those measured by the previous method which monitors flow-switching phenomena in the H-shaped microfluidic device. To induce flow-switching phenomena without occlusion in the bridge channel, the formation of platelet aggregation is minimized by anticoagulating blood samples with EDTA, which has significantly low *I*_*A·T*_, as depicted in Fig. 5C. Blood viscosities measured by the proposed method are well matched up with those measured by the previous method (Fig. 5D). Pearson’s linear correlation coefficient between them is about 0.98. Measurement accuracy of the proposed method for quantifying platelet adhesion is checked by comparing the correlation maps to the corresponding optical images. Blood samples anticoagulated by heparin at different concentration *C*_*Heparin*_ of 100 and 300 IU/mL are delivered into the microchannel via the peristaltic pump. To obtain optical images of adhered platelets, blood inside the microfluidic device is carefully washed away with PBS solution. As shown in Fig. 5E, the correlation maps and optical images for both concentration conditions are matched up well. Similar to Fig. 5C, the area of adhered platelets for *C*_*Heparin*_ = 100 IU/mL is much higher than that for *C*_*Heparin*_ = 100 IU/mL (*A*_*Platelet*_ = 0.25 and 0.16 for *C*_*Heparin*_ = 100 and 300 IU/mL). The correlation coefficient between the correlation maps and optical images are 0.63 and 0.55 for *C*_*Heparin*_ = 100 and 300 IU/mL, respectively. The slightly low correlation coefficient may be associated with the detachment and shape change of adhered platelets due to injection of PBS solution. These validation results demonstrate that the proposed method can be used to measure blood viscosity and platelet adhesion reasonably.

### *Ex vivo* measurement

**Figure 6:**
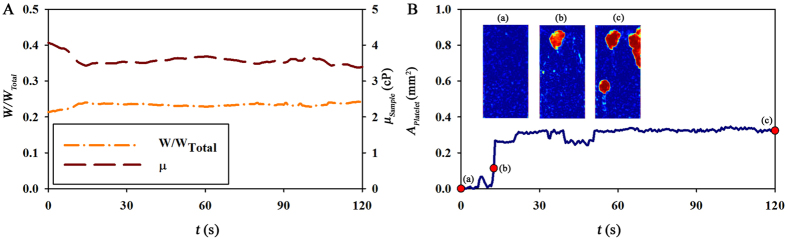
*Ex vivo* measurement by using a rat extracorporeal model. Temporal variations in the (**A**) *W/W*_*Total*_ and *μ*_*Sample*_, as well as (**B**) *I*_*A·T*_ obtained under *ex vivo* condition. Typical correlation maps at specific times of (a–c) (t = 0, 12.5, and 120 s) are inserted.

By measuring the width ratio (*W*/*W*_*Total*_) and the platelet area *A*_*Platelet*_, blood viscosity and platelet function can be simultaneously estimated under *ex vivo* conditions. *ω*_*Speed*_ is set to be 3. Figure 6D,E show temporal variations in the *W*/*W*_*Total*_, *μ*_*Blood*_ and *A*_*Platelet*_ for representative normal rat. The mean value of *μ*_*Blood*_ is 3.71 cP. The blood viscosity measured in the present study is relatively lower than that measured in previous *ex vivo* measurements^38^,^39^. Considering the shear thinning effect of the blood, the relatively low blood viscosity has some relation with high shear rate in this study (60,000 s^−1^). Arterial blood pressure can induce discrepancy between the actual and estimated flow rates. This increase of flow rate due to arterial pressure is removed by manipulating the pinch valve to prevent inaccurate measurement of blood viscosity. As depicted in the insets of Fig. 6E[a–c], large platelet aggregates adhere to the channel more rapidly. As time goes by, this adhesion of platelets becomes considerable with time.

However, this method has some technological limitations. The measured viscosity becomes slightly inaccurate, when platelets activated by the peristaltic pump stick around the interfacial line. On average, the measurement takes about 2 min. Although the measurement time is relatively short, PBS solution of about 0.33 mL is injected into the vein of the rat model during the measurement. This injection can lead to hemodilution. Moreover, it is difficult for the present method to repeat similar experiments using the same device, because the platelets adhered in previous experiment might change the probability of platelet adhesion significantly. Considering that the platelets adhered to the collagen film make leukocytes adhere to them under blood flow condition^51^, the level of platelet adhesion measured by the proposed method may be slightly overestimated due to the adhesion of leukocytes. Since leukocytes do not significantly adhere by themselves^52^, we can infer that the degree of overestimation may not be so significant. For more accurate analysis, additional modification of the measurement system, and study on leukocyte adhesion are required in the near future.

### Comparison between normal and diabetic samples

Glucose concentration of blood is measured using an Accu-Chek® Sensor instrument with test strips (Roche Diagnostics, Mannheim, Germany) after the experiment. As summarized in Table 1, the glucose concentration of diabetic rats is about 3.5 times larger than that of the control rats. The weight of normal rats is slightly high compared to diabetic rats. Through the *ex vivo* measurements, *t*_*Blood*_ and *I*_*A·T*_ for two groups are obtained. *μ*_*Blood*_ and *I*_*A·T*_ of diabetic rats are relatively higher than those of the control rats. These results are in accordance with previous studies conducted on STZ-induced rats^53^,^54^. It may be caused by increase in plasma protein such as soluble thrombomodulin (sTM), von Willebrand factor (vWF) and fibrinogen^55^. Based on these results, we concluded that the proposed method can accurately detect the variation of blood viscosity with measurement accuracy of about 0.073 cP and platelet adhesion with resolution of about 12 μm^2^·s.

## Conclusion

In the present study, platelet adhesion and blood viscosity are simultaneously measured by modifying our previous microfluidic-based method. Specifically, blood samples flow with a peristaltic pump. After passing through a tube inside the peristaltic pump, platelet aggregates are formed because rotors inside the peristaltic pump make high shear condition. These platelet aggregates adhere to the microfluidic device under blood flow conditions. By applying the correlation mapping method, the area of adhered platelets (*A*_*Platelet*_) can be easily estimated. To estimate the platelet function, representative index (*I*_*A·T*_) is determined based on *A*_*Platelet*_ and the contact time. In order to measure the viscosity, flow conditions in the H-shaped device are continuously monitored. By blocking the outlet of the reference flow, the reference fluid (a PBS solution) and samples (PRP, blood samples) flow into one side channel. Because the interfacial width (*W*) of PBS solution in the side channel can be determined by the pressure ratio of the reference to the sample flows, a fitting equation depicting the relation between pressure ratio and interfacial width is obtained from the experiments with PBS solutions. Therefore, the pressure ratio can be estimated by applying the interfacial width ratio (*W/W*_*Total*_) measured by image processing to the fitting equation (Equation 4). Based on the pressure ratio, viscosity of samples (*μ*) can be measured under given flow conditions. The feasibility of the proposed method for simultaneous measurements of platelet function and blood viscosity is demonstrated by using PRP and blood samples. Finally, variations in biophysical parameters between normal and diabetic rats are compared under *ex vivo* condition using a rat extracorporeal model. The present estimation method is potentially useful as a complementary diagnostic modality in evaluating the biophysical properties of animal model with cardiovascular diseases without the need for blood collection.

## Materials

### Fabrication of microfluidic device

The PDMS microfluidic device was fabricated using the photolithography replica molding process. A 4-inch silicon wafer was spin-coated with SU8 photo-resist. The SU8 film was exposed to ultraviolet light through a mask pattern positioned on a mask aligner. The unexposed part of the photoresist was washed away during the development process, and only the microfabricated template was left. Then, the silicon wafer was etched by Deep Reactive Ion (DRI) etching to form microchannel of 50μm in depth and the remained photoresist was washed away. PDMS prepolymer (Sylgard 184; Dow Corning, USA) was mixed with a curing reagent at a mass ratio of 10:1. The mixture was poured onto the mold. Air bubbles trapped in the PDMS were removed in a vacuum chamber for 1 h. Then, it is cured at 80 °C for 3 h. The PDMS block is peeled off from the silicon mold. The side channels of the H-shaped device have a width of 1 mm and a length of 10 mm. Each side channels has its own inlet and outlet made by a 1mm diameter puncher. After oxygen-plasma treatment (CUTE, Femto Science, Korea) of 40 W with a flow rate of 30 sccm at a base pressure of 0.923 Torr for 90 s, the microfluidic device is finally prepared by bonding the PDMS block to a glass substrate. Platelet adhesion is facilitated by coating the glass substrate with collagen^56^.

### Blood sample preparation

Blood samples are collected from male Sprague–Dawley (SD) rats (12 weeks old) through abdominal aortic puncture under intramuscular anesthesia with ketamine (100 mg/kg) and xylazine (10 mg/kg). The sample are anticoagulated with ethylenediaminetetraacetic acid (EDTA) dipotassium salt (1.5 mg of EDTA per 1 mL of blood). A platelet-rich plasma (PRP) is obtained by centrifuging whole blood at 500 g for 6 minutes. The separated RBCs are carefully remixed with the PRP to have specific hematocrit levels (10, 30, and 50%) for investigating the effect of hematocrit on platelet adhesion. The variations in hemorheological properties caused by heparin are evaluated by comparing the results for blood anticoagulated with EDTA to those for blood anticoagulated with heparin solution (1000 IU/mL). The concentrations of blood anticoagulated with heparin are fixed as 100 and 300 IU/mL. Each experiment is repeated at least three times by using different samples. Experiments are completed within 1 h from the blood collection to prevent remarkable variations in hemorheological properties. All experimental procedures performed on the animals were carried out in accordance with the approved guidelines of Ethics Committee of POSTECH.

### Experimental setup

The microfluidic device is observed on an optical microscope (Nikon, Tokyo, Japan) with 4× objective lens (NA of 0.1). Four frames of flow in the microfluidic device are consecutively captured by a high-speed camera (FASTCAM SA 1.1, Photron Ltd., San Diego, USA) at 5000 fps, when a trigger signal generated by a delay generator (model 555, BNC, USA) was input to the camera. The time interval between every two trigger signals is 0.5 s. The detailed procedure of image acquisition is described in our previous study^57^.

### Preparation of diabetic rats

To induce type 1 diabetes with hyperglycemia, SD rats (12 weeks old) are treated via intraperitoneal injection of STZ (65 mg/kg in sterile saline) under anesthesia with isoflurane and oxygen. The detailed procedure used in the present study is well described in our previous study^57^. After ten days from the treatment, rat models are used for *ex vivo* measurement.

### Preparation of rat extracorporeal model

Normal and diabetic rat samples (14 weeks old; n = 2) are anesthetized with intramuscular injection of ketamine (100 mg/kg) and xylazine (10 mg/kg). As shown in Fig. 1A, a bypass loop is composed of PE-50 (ID = 0.58 mm, polyethylene tube) tube, a flow stabilization device with an air cavity of 0.5 mL and microchannel. The PE-50 tube at one end of the bypass loop is cannulated into the right jugular vein. A 1500 IU/mL/kg of heparin is injected into the jugular vein to prevent blood clotting in the bypass loop. After 10 min of heparin injection, the PE-50 tube at the other end of the bypass loop is cannulated into the femoral artery of the rat sample. The blood supplied from artery is collected in a chamber of 2 mL in volume. By manipulating the pinch valve connected with PE-50 tube between the artery vessel and the chamber, blood sample is stably supplied into the left side channel of the microfluidic device by a peristaltic pump (MP-1000; Tokyo Rikakikai CO., Ltd., Tokyo, Japan) with a Tygon tube (diameter: 510 μm; wall thickness: 500 μm). A phosphate-buffered saline (PBS; pH 7.4, Bio Solution, Korea) solution is supplied into the right side channel by a syringe pump (neMESYS, Centoni Gmbh, Germany). A mixture of blood and PBS solution is returned to the jugular vein of the rat model. The Animal Care and Ethics Committee of POSTECH approved all the procedures performed on the animals. All experiments were carried out in accordance with the approved guidelines.

## Additional Information

**How to cite this article**: Yeom, E. *et al.* Microfluidics for simultaneous quantification of platelet adhesion and blood viscosity. *Sci. Rep.* **6**, 24994; doi: 10.1038/srep24994 (2016).

## Supplementary Material

Supplementary Information

## Figures and Tables

**Table 1:** Comparison of various indices between normal and diabetic rat samples.

	Normal rat	Diabetic rat
Blood sugar (mg/dL)	111.9 ± 9.6	389.5 ± 34.7
Weight (g)	343.4 ± 2.3	301.1 ± 2.7
*μ* (cP)	3.26 ± 0.28	4.23 ± 0.31
*I*_*A·T*_ (mm^2^·s)	24.3 ± 9.6	58.3 ± 10.3

Each value represents mean ± standard deviation.
