# Supplementary material for: Microfluidics for simultaneous quantification of platelet adhesion and blood viscosity
Source: Sci Rep. 2016 Apr 27;6:24994. doi: 10.1038/srep24994 (PMC4846989; doi:10.1038/srep24994)
Supplement: Supplementary Information [file srep24994-s1.pdf]

# Microfluidics for simultaneous quantification of platelet adhesion and blood viscosity

Eunseop Yeom[1], Jun Hong Park[2], Yang Jun Kang[3] and Sang Joon Lee[2*]

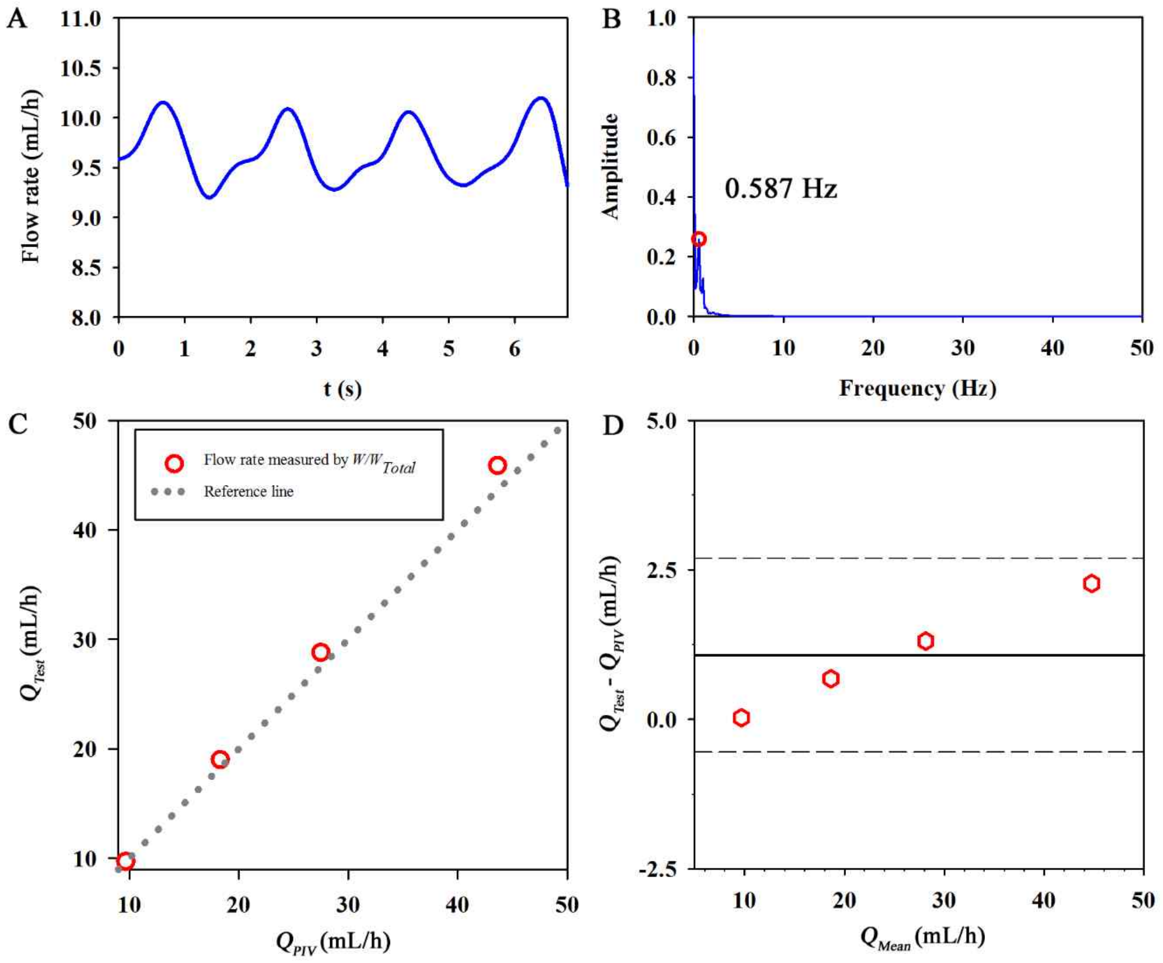


**Supplementary Fig. 1 (A)** Temporal variation of flow rate at $\omega_{Speed}$ =3. To measure the flow rate, a micro-PIV technique is applied to flow images acquired at 5000 fps with a time interval of 0.01 s. **(B)** FFT (Fast Fourier transform) analysis result for the flow rate at $\omega_{Speed}$ =3. Red circle indicates the peak value at 0.587 Hz. **(C)** Comparison of flow rates measured by the proposed method ($Q_{Test}$) and micro-PIV technique ($Q_{PIV}$) at different $\omega_{Speed}$ values. **(D)** Difference between the measured flow rates ($Q_{Test} - Q_{PIV}$) is depicted in the Bland–Altman plot with respect to the average value ($Q_{Mean}$). A bold line and dashed lines denote the mean value and 95% coverage intervals, respectively.
